# Dichlorido(9-methyl­adenine-κ*N* ^7^)(η^5^-penta­methyl­cyclo­penta­dien­yl)iridium(III) dichloromethane solvate

**DOI:** 10.1107/S1600536808003760

**Published:** 2008-02-06

**Authors:** Clemens Bruhn, Thomas Küger, Dirk Steinborn

**Affiliations:** a Universität Kassel, FB 18, Naturwissenschaften, Abt. Metallorganische Chemie, Heinrich-Plett-Strasse 40, 34132 Kassel, Germany; b Martin-Luther-Universität Halle-Wittenberg, Institut für Chemie–Anorganische Chemie, Kurt-Mothes-Strasse 2, 06120 Halle, Germany

## Abstract

In the title complex, [Ir(C_10_H_15_)Cl_2_(C_6_H_7_N_5_)]·CH_2_Cl_2_ or [Ir(η^5^-C_5_Me_5_)Cl_2_(9-MeAde-κ*N* ^7^)]·CH_2_Cl_2_ (9-MeAde = 9-methyl­adenine), the coordination geometry of the Ir^III^ atom approximates to a three-legged piano stool. The 9-methyl­adenine ligand is coordinated in a monodentate fashion to the Ir centre through its N-7 atom. The crystal structure contains centrosymmetric pairs of mol­ecules, inter­acting through two N—H⋯N hydrogen bonds. An intra­molecular N—H⋯Cl hydrogen bond is formed between the H atom of an NH_2_ group and a chlorido ligand. Further short intra- and inter­molecular C—H⋯Cl contacts are observed.

## Related literature

For background information, see: Lippert (2000 ▶); Houlton (2002 ▶). For related literature, see: Zhu *et al.* (2002 ▶); Gaballa *et al.* (2004 ▶, 2008 ▶); Aakeröy *et al.* (1999 ▶); Baldovino-Pantaleon *et al.* (2007 ▶); Davies *et al.* (2003 ▶); Huang *et al.* (1998 ▶); Jeffrey & Saenger (1994 ▶); Kistenmacher & Rossi (1977 ▶); McMullan *et al.* (1980 ▶).
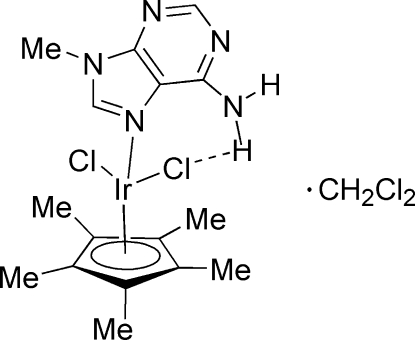


## Experimental

### 

#### Crystal data

[Ir(C_10_H_15_)Cl_2_(C_6_H_7_N_5_)]·CH_2_Cl_2_
*M* *_r_* = 632.41
Triclinic, 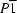

*a* = 7.294 (2) Å
*b* = 11.8698 (14) Å
*c* = 13.649 (3) Å
α = 71.338 (15)°
β = 83.83 (3)°
γ = 78.003 (14)°
*V* = 1094.0 (4) Å^3^
*Z* = 2
Mo *K*α radiation
μ = 6.60 mm^−1^
*T* = 200 (2) K
0.19 × 0.15 × 0.13 mm

#### Data collection

Stoe STADI-4 diffractometer
Absorption correction: multi-scan (*X-RED*; Stoe & Cie, 2002 ▶) *T*_min_ = 0.32, *T*_max_ = 0.43
4132 measured reflections
3807 independent reflections
3246 reflections with *I* > 2σ(*I*)
*R*_int_ = 0.068
1 standard reflections frequency: 60 min intensity decay: none

#### Refinement

*R*[*F* ^2^ > 2σ(*F* ^2^)] = 0.047
*wR*(*F* ^2^) = 0.125
*S* = 1.13
3807 reflections
250 parameters
H-atom parameters constrained
Δρ_max_ = 2.87 e Å^−3^
Δρ_min_ = −3.41 e Å^−3^

### 

Data collection: *STADI4* (Stoe & Cie, 2002 ▶); cell refinement: *STADI4* (Stoe & Cie, 2002 ▶); data reduction: *X-RED* (Stoe & Cie, 2002 ▶); program(s) used to solve structure: *SHELXS97* (Sheldrick, 2008 ▶); program(s) used to refine structure: *SHELXL97* (Sheldrick, 2008 ▶); molecular graphics: *DIAMOND* (Brandenburg, 1999 ▶); software used to prepare material for publication: *SHELXL97*.

## Supplementary Material

Crystal structure: contains datablocks I, global. DOI: 10.1107/S1600536808003760/fj2096sup1.cif

Structure factors: contains datablocks I. DOI: 10.1107/S1600536808003760/fj2096Isup2.hkl

Additional supplementary materials:  crystallographic information; 3D view; checkCIF report

## Figures and Tables

**Table:** 

C10—Ir	2.127 (10)
C11—Ir	2.165 (10)
C12—Ir	2.164 (10)
C13—Ir	2.159 (11)
C14—Ir	2.153 (10)
Cl1—Ir	2.402 (3)
Cl2—Ir	2.423 (3)
N7—Ir	2.152 (8)

**Table:** 

N7—Ir—Cl1	86.0 (2)
N7—Ir—Cl2	91.0 (2)
Cl1—Ir—Cl2	85.72 (9)

**Table 2:** Hydrogen-bond geometry (Å, °)

*D*—H⋯*A*	*D*—H	H⋯*A*	*D*⋯*A*	*D*—H⋯*A*
N6—H6*A*⋯N1^i^	0.88	2.14	3.007 (13)	170
N6—H6*B*⋯Cl2	0.88	2.35	3.168 (10)	155
C8—H8⋯Cl1	0.95	2.77	3.237 (11)	111
C8—H8⋯Cl1^ii^	0.95	2.65	3.537 (11)	156
C9—H9*B*⋯Cl3^iii^	0.98	2.75	3.697 (13)	163
C20—H20*B*⋯Cl1^ii^	0.99	2.75	3.519 (15)	135

Symmetry codes: (i) 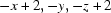; (ii) 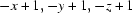; (iii) 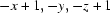.

**Fig. 1.**
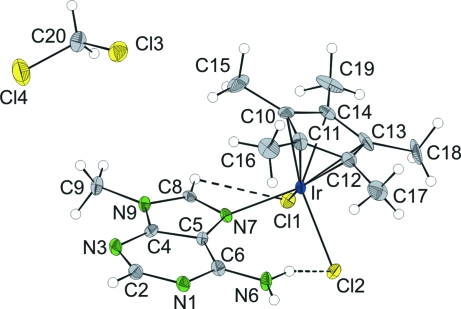
Structure of the asymmetric unit of the title complex [IrCl2(η5-C5Me5)(9-MeAde-κN7)].CH2Cl2. Displacement ellipsoids are drawn at the 30% probability level and H atoms are shown as small spheres of arbitrary radii.

**Fig. 2.**
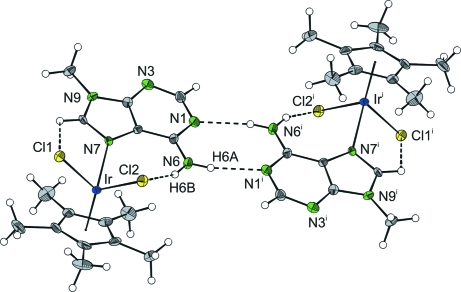
Structure of the dinuclear complex [{IrCl2(η5-C5Me5)(9-MeAde-κN7)}2] in crystals of the title compound. Displacement ellipsoids are drawn at the 30% probability level and H atoms are shown as small spheres of arbitrary radii. The numbering scheme of the C atoms is as shown in Figure 1. Symmetry codes: (i) –x + 2, –y, –z + 2.

## supplementary crystallographic information

### Comment

Due to their importance in chemotherapy, nucleobase complexes of platinum and other transition metals attract attention. We are interested in syntheses and characterization of such complexes having, especially, metals in higher oxidation states (Zhu *et al.*, 2002; Gaballa *et al.*, 2004; Gaballa *et al.*, 2007). The iridium(III) title complex [IrCl_2_(η^5^-C_5_Me_5_)(9-MeAde-κ*N*^7^)].CH_2_Cl_2_ (see Figure 1) crystallizes in the triclinic space group *P*1. Crystals contain centrosymmetric dinuclear molecules (see Figure 2). The coordination geometry of the iridium center approximates a three-legged piano stool, the irdium atom being directly bound to two chloro ligands, to a *N*7 coordinated 9-methyladenine ligand and to a η^5^-pentamethylcyclopentadienyl ligand. The 9-MeAde ligand is planar in good approximation, the greatest deviation from the mean plane was found for the exocyclic N6 atom (0.06 (1) Å). The Ir–N7 and Ir–Cl1/Ir–Cl2 bonds are as long as those in the complex [IrCl_2_(η^5^-C_5_Me_5_)(NH_2_Ph-κ*N*)] (2.152 (8) *versus*. 2.152 Å and 2.402 (3)/2.423 (3) *versus*. 2.394/2.419 Å) (Davies *et al.*, 2003).

The dimers are formed through two N6–H6A···N1' hydrogen bonds (N6···N1' 3.01 (1) Å; H6A···N1' 2.14 Å; N6–H6A···N1' 170°). Furthermore, the other hydrogen atom of the exocyclic amino group acts as hydrogen donor in a N6–H6B···Cl2 hydrogen bond (N6···Cl2 3.17 (1) Å; H6B···Cl2 2.35 Å; N6–H6B···Cl2 155°). The structural parameters of these two hydrogen bonds are in accord with analogous hydrogen bonds in nucleobases and in chloro metal complexes, respectively (Jeffrey & Saenger, 1994; Baldovino-Pantaleon *et al.*, 2007). Noteworthy, in crystals of 9-methyladenine two N6–H6A···N1' and N6–H6B···N7' hydrogen bonds link molecules in ribbons (Kistenmacher & Rossi, 1977; McMullan *et al.*, 1980). Furthermore, short intra- and intermolecular C–H···Cl contacts (see Table) indicate stabilizing interactions (Huang *et al.*, 1998; Aakeröy *et al.*, 1999).

### Experimental

Reaction of [{IrCl_2_(η^5^-C_5_Me_5_)}_2_] with 9-methyladenine (9-MeAde) in 1: 2 ratio in methylene chloride resulted in the formation of yellow crystals of the title complex in 67% yield. ^1^H NMR (CD_2_Cl_2_, 200 MHz): δ 1.49 (s, 15H, C_5_(C*H*_3_)_5_), 3.88 (s, 3H, NC*H*_3_), 8.41 (s, br, 1H, *H*8), 8.64 (s, br, 1H, *H*2).

### Refinement

All non-H atoms were refined with anisotropic thermal parameters. H atoms were included in the model in calculated positions using the riding model, with their isotropic displacement parameter tied to 1.2 times that of the bonded atom.

### Crystal data

**Table:**

[Ir(C_10_H_15_)Cl_2_(C_6_H_7_N_5_)]·CH_2_Cl_2_	*Z* = 2
*M**_r_* = 632.41	*F*(000) = 612
Triclinic, *P*1	*D*_x_ = 1.920 Mg m^−^^3^
Hall symbol: -P 1	Mo *K*α radiation, λ = 0.71073 Å
*a* = 7.294 (2) Å	Cell parameters from 32 reflections
*b* = 11.8698 (14) Å	θ = 6.5–18.9°
*c* = 13.649 (3) Å	µ = 6.60 mm^−^^1^
α = 71.338 (15)°	*T* = 200 K
β = 83.83 (3)°	Block, colourless
γ = 78.003 (14)°	0.19 × 0.15 × 0.13 mm
*V* = 1094.0 (4) Å^3^	

### Data collection

**Table:**

Stoe STADI-4 diffractometer	3246 reflections with *I* > 2σ(*I*)
Radiation source: fine-focus sealed tube	*R*_int_ = 0.068
graphite	θ_max_ = 25.0°, θ_min_ = 1.6°
profile data from ω/2θ scans	*h* = −8→8
Absorption correction: multi-scan (*X-RED*; Stoe & Cie, 2002)	*k* = −13→14
*T*_min_ = 0.32, *T*_max_ = 0.43	*l* = −8→16
4132 measured reflections	1 standard reflections every 60 min
3807 independent reflections	intensity decay: none

### Refinement

**Table:**

Refinement on *F*^2^	Primary atom site location: structure-invariant direct methods
Least-squares matrix: full	Secondary atom site location: difference Fourier map
*R*[*F*^2^ > 2σ(*F*^2^)] = 0.047	Hydrogen site location: inferred from neighbouring sites
*wR*(*F*^2^) = 0.125	H-atom parameters constrained
*S* = 1.13	*w* = 1/[σ^2^(*F*_o_^2^) + (0.0666*P*)^2^] where *P* = (*F*_o_^2^ + 2*F*_c_^2^)/3
3807 reflections	(Δ/σ)_max_ < 0.001
250 parameters	Δρ_max_ = 2.87 e Å^−^^3^
0 restraints	Δρ_min_ = −3.41 e Å^−^^3^
0 constraints	

### Special details

**Table:**

Geometry. All e.s.d.'s (except the e.s.d. in the dihedral angle between two l.s. planes) are estimated using the full covariance matrix. The cell e.s.d.'s are taken into account individually in the estimation of e.s.d.'s in distances, angles and torsion angles; correlations between e.s.d.'s in cell parameters are only used when they are defined by crystal symmetry. An approximate (isotropic) treatment of cell e.s.d.'s is used for estimating e.s.d.'s involving l.s. planes.
Refinement. Refinement of *F*^2^ against ALL reflections. The weighted *R*-factor *wR* and goodness of fit *S* are based on *F*^2^, conventional *R*-factors *R* are based on *F*, with *F* set to zero for negative *F*^2^. The threshold expression of *F*^2^ > σ(*F*^2^) is used only for calculating *R*-factors(gt) *etc*. and is not relevant to the choice of reflections for refinement. *R*-factors based on *F*^2^ are statistically about twice as large as those based on *F*, and *R*- factors based on ALL data will be even larger.

### Fractional atomic coordinates and isotropic or equivalent isotropic displacement parameters (Å^2^)

**Table:**

	*x*	*y*	*z*	*U*_iso_*/*U*_eq_	
C2	0.9961 (15)	−0.0640 (10)	0.7978 (9)	0.030 (2)	
H2	1.0628	−0.1450	0.8165	0.036*	
C4	0.8452 (14)	0.0945 (9)	0.6832 (8)	0.023 (2)	
C5	0.7931 (13)	0.1571 (8)	0.7549 (7)	0.019 (2)	
C6	0.8617 (15)	0.0991 (9)	0.8549 (8)	0.026 (2)	
C8	0.6814 (13)	0.2726 (9)	0.6092 (8)	0.020 (2)	
H8	0.6198	0.3402	0.5573	0.024*	
C9	0.7869 (16)	0.1435 (10)	0.4929 (8)	0.029 (2)	
H9A	0.7137	0.2109	0.4416	0.035*	
H9B	0.7375	0.0696	0.5034	0.035*	
H9C	0.9185	0.1318	0.4682	0.035*	
C10	0.2376 (14)	0.3257 (10)	0.7454 (8)	0.028 (2)	
C11	0.3065 (16)	0.2573 (10)	0.8485 (9)	0.030 (3)	
C12	0.2955 (15)	0.3417 (11)	0.9036 (8)	0.031 (3)	
C13	0.2174 (16)	0.4615 (11)	0.8368 (10)	0.037 (3)	
C14	0.1805 (14)	0.4482 (10)	0.7419 (9)	0.030 (3)	
C15	0.213 (2)	0.2713 (14)	0.6624 (11)	0.053 (4)	
H15A	0.2482	0.3242	0.5945	0.064*	
H15B	0.0821	0.2632	0.6635	0.064*	
H15C	0.2941	0.1914	0.6754	0.064*	
C16	0.3688 (18)	0.1234 (10)	0.8885 (11)	0.045 (3)	
H16A	0.4789	0.1048	0.9301	0.054*	
H16B	0.4014	0.0907	0.8301	0.054*	
H16C	0.2671	0.0869	0.9315	0.054*	
C17	0.3513 (19)	0.3108 (14)	1.0118 (9)	0.050 (4)	
H17A	0.4030	0.3772	1.0197	0.060*	
H17B	0.4465	0.2366	1.0282	0.060*	
H17C	0.2412	0.2982	1.0591	0.060*	
C18	0.180 (2)	0.5756 (12)	0.8658 (13)	0.060 (4)	
H18A	0.2126	0.6420	0.8067	0.072*	
H18B	0.2555	0.5651	0.9243	0.072*	
H18C	0.0464	0.5946	0.8855	0.072*	
C19	0.0889 (17)	0.5477 (13)	0.6515 (12)	0.056 (4)	
H19A	0.0830	0.6263	0.6621	0.068*	
H19B	−0.0383	0.5361	0.6462	0.068*	
H19C	0.1627	0.5454	0.5876	0.068*	
C20	0.331 (2)	0.2130 (12)	0.3148 (10)	0.045 (3)	
H20A	0.2167	0.2111	0.2826	0.053*	
H20B	0.3533	0.2972	0.2903	0.053*	
Cl1	0.5584 (4)	0.5480 (2)	0.6167 (2)	0.0268 (5)	
Cl2	0.7133 (4)	0.4366 (2)	0.8520 (2)	0.0289 (6)	
Cl3	0.2938 (5)	0.1691 (3)	0.4491 (3)	0.0505 (8)	
Cl4	0.5213 (6)	0.1194 (4)	0.2750 (4)	0.0745 (12)	
N1	0.9631 (12)	−0.0139 (8)	0.8738 (7)	0.027 (2)	
N3	0.9494 (13)	−0.0174 (7)	0.6998 (7)	0.029 (2)	
N6	0.8322 (13)	0.1512 (8)	0.9311 (7)	0.031 (2)	
H6A	0.8789	0.1115	0.9921	0.037*	
H6B	0.7663	0.2249	0.9199	0.037*	
N7	0.6850 (11)	0.2692 (7)	0.7069 (6)	0.0188 (17)	
N9	0.7732 (12)	0.1709 (8)	0.5912 (6)	0.0233 (18)	
Ir	0.47641 (5)	0.39087 (3)	0.76639 (3)	0.01833 (15)	

### Atomic displacement parameters (Å^2^)

**Table:**

	*U*^11^	*U*^22^	*U*^33^	*U*^12^	*U*^13^	*U*^23^
C2	0.030 (6)	0.026 (6)	0.033 (6)	0.002 (5)	−0.006 (5)	−0.011 (5)
C4	0.025 (5)	0.024 (5)	0.026 (5)	−0.008 (4)	−0.003 (4)	−0.011 (4)
C5	0.022 (5)	0.017 (5)	0.017 (5)	−0.004 (4)	0.001 (4)	−0.006 (4)
C6	0.028 (6)	0.023 (5)	0.021 (5)	0.002 (4)	−0.003 (4)	−0.003 (4)
C8	0.014 (5)	0.017 (5)	0.025 (5)	0.002 (4)	−0.004 (4)	−0.004 (4)
C9	0.043 (7)	0.025 (6)	0.022 (5)	−0.009 (5)	0.006 (5)	−0.011 (4)
C10	0.018 (5)	0.040 (7)	0.029 (6)	−0.016 (5)	0.005 (4)	−0.011 (5)
C11	0.033 (6)	0.025 (6)	0.035 (6)	−0.017 (5)	0.016 (5)	−0.009 (5)
C12	0.026 (6)	0.043 (7)	0.025 (6)	−0.013 (5)	0.010 (4)	−0.012 (5)
C13	0.023 (6)	0.030 (6)	0.053 (8)	0.005 (5)	0.012 (5)	−0.015 (6)
C14	0.018 (5)	0.028 (6)	0.037 (6)	0.006 (4)	−0.002 (4)	−0.004 (5)
C15	0.055 (9)	0.069 (10)	0.053 (9)	−0.046 (8)	0.006 (7)	−0.026 (8)
C16	0.048 (8)	0.015 (6)	0.064 (9)	−0.014 (5)	0.005 (6)	0.001 (6)
C17	0.041 (7)	0.074 (10)	0.029 (7)	−0.006 (7)	0.006 (6)	−0.013 (7)
C18	0.054 (9)	0.041 (8)	0.092 (12)	0.003 (7)	0.027 (8)	−0.047 (8)
C19	0.019 (6)	0.057 (9)	0.068 (10)	−0.001 (6)	0.000 (6)	0.012 (7)
C20	0.054 (8)	0.037 (7)	0.047 (8)	−0.007 (6)	−0.001 (6)	−0.020 (6)
Cl1	0.0323 (14)	0.0227 (13)	0.0256 (13)	−0.0081 (10)	−0.0010 (10)	−0.0057 (10)
Cl2	0.0349 (14)	0.0274 (13)	0.0295 (14)	−0.0074 (11)	−0.0051 (11)	−0.0136 (11)
Cl3	0.055 (2)	0.0467 (19)	0.058 (2)	−0.0206 (16)	0.0060 (16)	−0.0227 (16)
Cl4	0.071 (3)	0.081 (3)	0.072 (3)	0.008 (2)	0.009 (2)	−0.042 (2)
N1	0.028 (5)	0.018 (4)	0.033 (5)	0.000 (4)	−0.002 (4)	−0.008 (4)
N3	0.032 (5)	0.015 (4)	0.040 (6)	0.001 (4)	0.000 (4)	−0.013 (4)
N6	0.043 (6)	0.023 (5)	0.023 (5)	0.011 (4)	−0.009 (4)	−0.011 (4)
N7	0.021 (4)	0.010 (4)	0.020 (4)	0.004 (3)	−0.002 (3)	−0.001 (3)
N9	0.029 (5)	0.022 (4)	0.019 (4)	−0.006 (4)	0.005 (3)	−0.008 (4)
Ir	0.0201 (2)	0.0154 (2)	0.0201 (2)	−0.00199 (14)	0.00201 (14)	−0.00814 (15)

### Geometric parameters (Å, °)

**Table:**

C2—N3	1.325 (14)	C13—C18	1.494 (17)
C2—N1	1.331 (14)	C13—Ir	2.159 (11)
C2—H2	0.9500	C14—C19	1.510 (16)
C4—N3	1.348 (13)	C14—Ir	2.153 (10)
C4—N9	1.375 (13)	C15—H15A	0.9800
C4—C5	1.386 (14)	C15—H15B	0.9800
C5—N7	1.392 (12)	C15—H15C	0.9800
C5—C6	1.410 (14)	C16—H16A	0.9800
C6—N1	1.349 (13)	C16—H16B	0.9800
C6—N6	1.349 (14)	C16—H16C	0.9800
C8—N7	1.325 (13)	C17—H17A	0.9800
C8—N9	1.335 (13)	C17—H17B	0.9800
C8—H8	0.9500	C17—H17C	0.9800
C9—N9	1.467 (13)	C18—H18A	0.9800
C9—H9A	0.9800	C18—H18B	0.9800
C9—H9B	0.9800	C18—H18C	0.9800
C9—H9C	0.9800	C19—H19A	0.9800
C10—C14	1.414 (16)	C19—H19B	0.9800
C10—C11	1.463 (16)	C19—H19C	0.9800
C10—C15	1.514 (17)	C20—Cl4	1.743 (13)
C10—Ir	2.127 (10)	C20—Cl3	1.745 (13)
C11—C12	1.419 (16)	C20—H20A	0.9900
C11—C16	1.493 (15)	C20—H20B	0.9900
C11—Ir	2.165 (10)	Cl1—Ir	2.402 (3)
C12—C13	1.458 (16)	Cl2—Ir	2.423 (3)
C12—C17	1.484 (16)	N6—H6A	0.8800
C12—Ir	2.164 (10)	N6—H6B	0.8800
C13—C14	1.413 (17)	N7—Ir	2.152 (8)
			
N3—C2—N1	129.5 (10)	C12—C17—H17A	109.5
N3—C2—H2	115.3	C12—C17—H17B	109.5
N1—C2—H2	115.3	H17A—C17—H17B	109.5
N3—C4—N9	127.0 (9)	C12—C17—H17C	109.5
N3—C4—C5	127.0 (10)	H17A—C17—H17C	109.5
N9—C4—C5	106.0 (9)	H17B—C17—H17C	109.5
C4—C5—N7	108.9 (8)	C13—C18—H18A	109.5
C4—C5—C6	116.4 (9)	C13—C18—H18B	109.5
N7—C5—C6	134.6 (9)	H18A—C18—H18B	109.5
N1—C6—N6	119.0 (9)	C13—C18—H18C	109.5
N1—C6—C5	117.6 (9)	H18A—C18—H18C	109.5
N6—C6—C5	123.4 (9)	H18B—C18—H18C	109.5
N7—C8—N9	112.9 (8)	C14—C19—H19A	109.5
N7—C8—H8	123.5	C14—C19—H19B	109.5
N9—C8—H8	123.5	H19A—C19—H19B	109.5
N9—C9—H9A	109.5	C14—C19—H19C	109.5
N9—C9—H9B	109.5	H19A—C19—H19C	109.5
H9A—C9—H9B	109.5	H19B—C19—H19C	109.5
N9—C9—H9C	109.5	Cl4—C20—Cl3	112.3 (8)
H9A—C9—H9C	109.5	Cl4—C20—H20A	109.2
H9B—C9—H9C	109.5	Cl3—C20—H20A	109.2
C14—C10—C11	107.9 (10)	Cl4—C20—H20B	109.2
C14—C10—C15	126.5 (11)	Cl3—C20—H20B	109.2
C11—C10—C15	125.3 (11)	H20A—C20—H20B	107.9
C14—C10—Ir	71.7 (6)	C2—N1—C6	119.0 (9)
C11—C10—Ir	71.5 (6)	C2—N3—C4	110.4 (9)
C15—C10—Ir	127.3 (8)	C6—N6—H6A	120.0
C12—C11—C10	107.0 (10)	C6—N6—H6B	120.0
C12—C11—C16	126.9 (11)	H6A—N6—H6B	120.0
C10—C11—C16	126.1 (11)	C8—N7—C5	104.9 (8)
C12—C11—Ir	70.8 (6)	C8—N7—Ir	119.3 (6)
C10—C11—Ir	68.7 (5)	C5—N7—Ir	132.2 (6)
C16—C11—Ir	127.4 (8)	C8—N9—C4	107.2 (8)
C11—C12—C13	108.4 (10)	C8—N9—C9	126.4 (9)
C11—C12—C17	125.0 (12)	C4—N9—C9	126.3 (9)
C13—C12—C17	126.6 (12)	C10—Ir—N7	97.3 (4)
C11—C12—Ir	70.9 (6)	C10—Ir—C14	38.6 (4)
C13—C12—Ir	70.1 (6)	N7—Ir—C14	130.7 (4)
C17—C12—Ir	125.7 (8)	C10—Ir—C13	65.1 (5)
C14—C13—C12	107.3 (10)	N7—Ir—C13	160.2 (4)
C14—C13—C18	127.1 (12)	C14—Ir—C13	38.3 (5)
C12—C13—C18	125.6 (13)	C10—Ir—C12	65.4 (4)
C14—C13—Ir	70.6 (6)	N7—Ir—C12	126.5 (4)
C12—C13—Ir	70.4 (6)	C14—Ir—C12	64.8 (4)
C18—C13—Ir	125.4 (9)	C13—Ir—C12	39.4 (4)
C13—C14—C10	109.4 (10)	C10—Ir—C11	39.9 (4)
C13—C14—C19	125.8 (12)	N7—Ir—C11	95.5 (4)
C10—C14—C19	124.7 (12)	C14—Ir—C11	65.2 (4)
C13—C14—Ir	71.1 (6)	C13—Ir—C11	65.3 (4)
C10—C14—Ir	69.7 (6)	C12—Ir—C11	38.3 (4)
C19—C14—Ir	126.8 (8)	C10—Ir—Cl1	112.7 (3)
C10—C15—H15A	109.5	N7—Ir—Cl1	86.0 (2)
C10—C15—H15B	109.5	C14—Ir—Cl1	93.1 (3)
H15A—C15—H15B	109.5	C13—Ir—Cl1	108.6 (3)
C10—C15—H15C	109.5	C12—Ir—Cl1	147.4 (3)
H15A—C15—H15C	109.5	C11—Ir—Cl1	152.5 (3)
H15B—C15—H15C	109.5	C10—Ir—Cl2	160.2 (3)
C11—C16—H16A	109.5	N7—Ir—Cl2	91.0 (2)
C11—C16—H16B	109.5	C14—Ir—Cl2	138.2 (3)
H16A—C16—H16B	109.5	C13—Ir—Cl2	103.0 (4)
C11—C16—H16C	109.5	C12—Ir—Cl2	95.2 (3)
H16A—C16—H16C	109.5	C11—Ir—Cl2	121.6 (3)
H16B—C16—H16C	109.5	Cl1—Ir—Cl2	85.72 (9)

### Hydrogen-bond geometry (Å, °)

**Table:**

*D*—H···*A*	*D*—H	H···*A*	*D*···*A*	*D*—H···*A*
N6—H6A···N1^i^	0.88	2.14	3.007 (13)	170.
N6—H6B···Cl2	0.88	2.35	3.168 (10)	155.
C8—H8···Cl1	0.95	2.77	3.237 (11)	111.
C8—H8···Cl1^ii^	0.95	2.65	3.537 (11)	156.
C9—H9B···Cl3^iii^	0.98	2.75	3.697 (13)	163.
C20—H20B···Cl1^ii^	0.99	2.75	3.519 (15)	135.

Symmetry codes: (i) −*x*+2, −*y*, −*z*+2; (ii) −*x*+1, −*y*+1, −*z*+1; (iii) −*x*+1, −*y*, −*z*+1.
